# Enterovirus71 (EV71) Utilise Host microRNAs to Mediate Host Immune System Enhancing Survival during Infection

**DOI:** 10.1371/journal.pone.0102997

**Published:** 2014-07-21

**Authors:** Yan Long Edmund Lui, Tuan Lin Tan, Wee Hong Woo, Peter Timms, Louise Marie Hafner, Kian Hwa Tan, Eng Lee Tan

**Affiliations:** 1 School of Biomedical Sciences, Faculty of Health, Queensland University of Technology, Brisbane, Queensland, Australia; 2 Institute of Health and Biomedical Innovation, Queensland University of Technology, Brisbane, Queensland, Australia; 3 School of Chemical and Life Sciences, Singapore Polytechnic, Singapore, Singapore; 4 Centre for Biomedical and Life Sciences, Singapore Polytechnic, Singapore, Singapore; 5 Faculty of Science, Health, Education and Engineering, University of the Sunshine Coast, Sippy Downs, Queensland, Australia; 6 Department of Paediatrics, University Children’s Medical Institute, National University Hospital, Singapore, Singapore; University of Hong Kong, Hong Kong

## Abstract

Hand, Foot and Mouth Disease (HFMD) is a self-limiting viral disease that mainly affects infants and children. In contrast with other HFMD causing enteroviruses, Enterovirus71 (EV71) has commonly been associated with severe clinical manifestation leading to death. Currently, due to a lack in understanding of EV71 pathogenesis, there is no antiviral therapeutics for the treatment of HFMD patients. Therefore the need to better understand the mechanism of EV71 pathogenesis is warranted. We have previously reported a human colorectal adenocarcinoma cell line (HT29) based model to study the pathogenesis of EV71. Using this system, we showed that knockdown of DGCR8, an essential cofactor for microRNAs biogenesis resulted in a reduction of EV71 replication. We also demonstrated that there are miRNAs changes during EV71 pathogenesis and EV71 utilise host miRNAs to attenuate antiviral pathways during infection. Together, data from this study provide critical information on the role of miRNAs during EV71 infection.

## Introduction

Hand, Foot and Mouth Disease (HFMD) is a contagious viral disease that commonly affects infants and children, with blisters and flu like symptoms [1]–[6]. HFMD is caused by a group of enteroviruses such as enterovirus 71 (EV71) and coxsackievirus A16 (CA16) [7]–[9]. However, unlike other HFMD causing enteroviruses, EV71 is also commonly associated with severe clinical diseases, including neurological diseases such as aseptic meningitis, brainstem and cerebellar encephalitis leading to cardiopulmonary failure and death [3], [10]–[13]. Due in part to a lack of understanding of viral pathogenesis of EV71 causing HFMD, there are no antiviral therapies or vaccines approved by the United States Food and Drug Administration (FDA) to prevent HFMD infections. There is therefore a need to gain a better understanding of the mechanism of EV71 pathogenesis. During infection, viruses attenuate host gene expression to enhance their survival [14]–[18]. Such activities include altering the cellular microenvironment to allow successful virus replication and evasion of the host immune system [19].

The host innate system is the first line of defence with the interferon (IFNs) being a key component against viral infection [17], [18], [20]–[25]. The release of IFNs begins with the recognition of pathogen-associated molecular patterns (PAMPs) which include Toll like receptors (TLRs), NOD like receptors (NLRs), retinoic acid-inducible gene I (RIG-I) like receptors [24], [26]. This family of cytokines act in an autocrine and paracrine manner to induce expression of transcription factors, gamma activated sequence and IFN stimulated response element and the consequent induction of IFN-stimulated genes (ISGs) which function to slow down viral infection [20]–[22]. Antiviral pathways such as the programmed cell death or apoptosis could also be activated by the host cells in attempt to control the infection by limiting viral replication [19].

Many viruses have evolved to counteract with strategies by host cells to establish infection. One of the mediators for such regulation includes genes that encode for microRNA (miRNAs). microRNAs are small single-stranded RNA species of approximately 20–24 bases in length that play a pivotal role in the post transcriptional regulation of gene expression [27], [28]. These small RNA are first discovered in *Caenorhabditis elegans* but was later discovered in a wide range of organisms from uni to multicellular eukaryotes [29]–[31]. miRNAs are found to regulate many cellular functions including cell proliferation, differentiation, homeostasis, immune activation and apoptosis [28], [29], [32]–[34]. As such, miRNAs can contribute to the repertoire of host-pathogen interactions during infection by modulating and directing host and viral gene expression thereby playing a pivotal role during infection. Elucidating the mechanism that the virus uses to bypass host defence systems and establish infections will provide us with a better understanding of the pathogenesis mechanism of the EV71 virus, allowing development of potent antiviral therapeutics for HFMD patients.

In this study, we explore the role of miRNAs in the pathogenesis of EV71 infected HT29 cells and identified key miRNA changes due to EV71 infection which might play a key role during EV71 pathogenesis.

## Results

We have previously reported a human colorectal adenocarcinoma cell line (HT29) with epithelioid morphology as a useful model to study the pathogenesis of EV71 [35]. Utilising the HT29 as a model, this study further elucidate the role of miRNAs during EV71 infection.

### Knockdown of DGCR8 resulted in the reduction of EV71 replication

**Figure 1:**
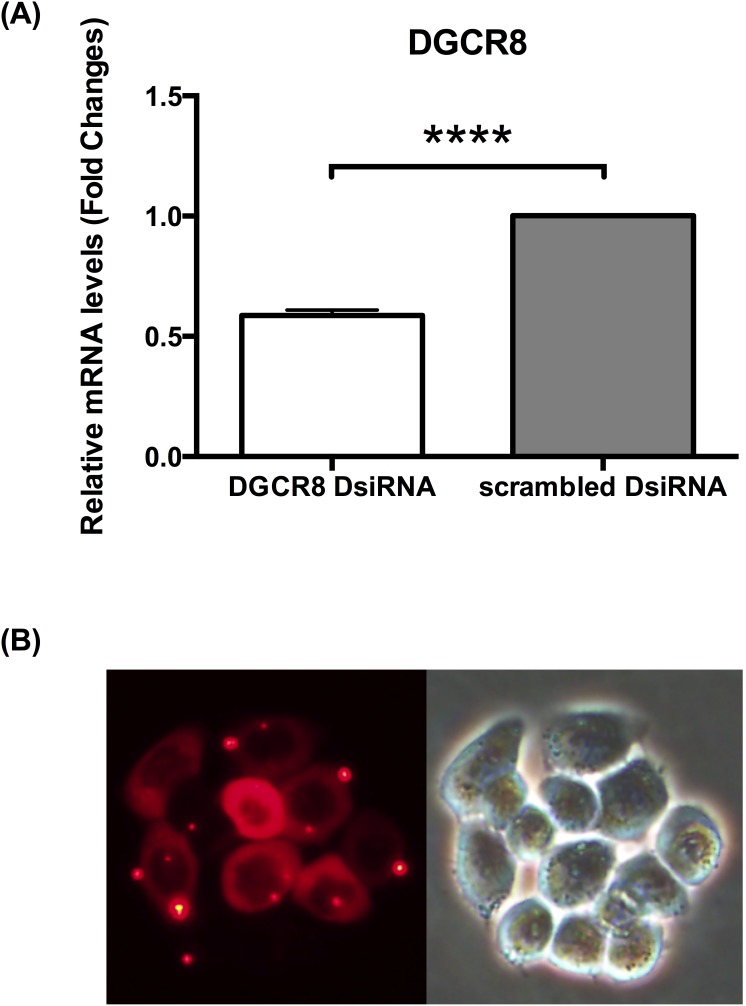
Knockdown of DGCR8 using DsiRNA. (A) Relative expression of DGCR8 gene measured by qPCR verifying knockdown. (n = 3, **** = *p* values of <0.0001) (B) Transfection Control using fluorescent-labeled transfection control duplex TYE 563 DS to monitor transfection efficiency.

In order to characterise the role of miRNAs during EV71 pathogenesis, DGCR8 was knocked down prior to infection. HT29 cells were transfected with DsiRNA specific to DGCR8, an essential cofactor for miRNAs biogenesis. As control, HT29 cells were transfected with NC1, negative control duplex-scrambled DsiRNA. Knockdown of DGCR8 was verified using qPCR (Figure 1a). Transfection was shown to be successful using TYE 563 DS Transfection Control, fluorescent-labelled transfection control duplex (Figure 1b).

### miRNAs is essential for EV71 replication

**Figure 2:**
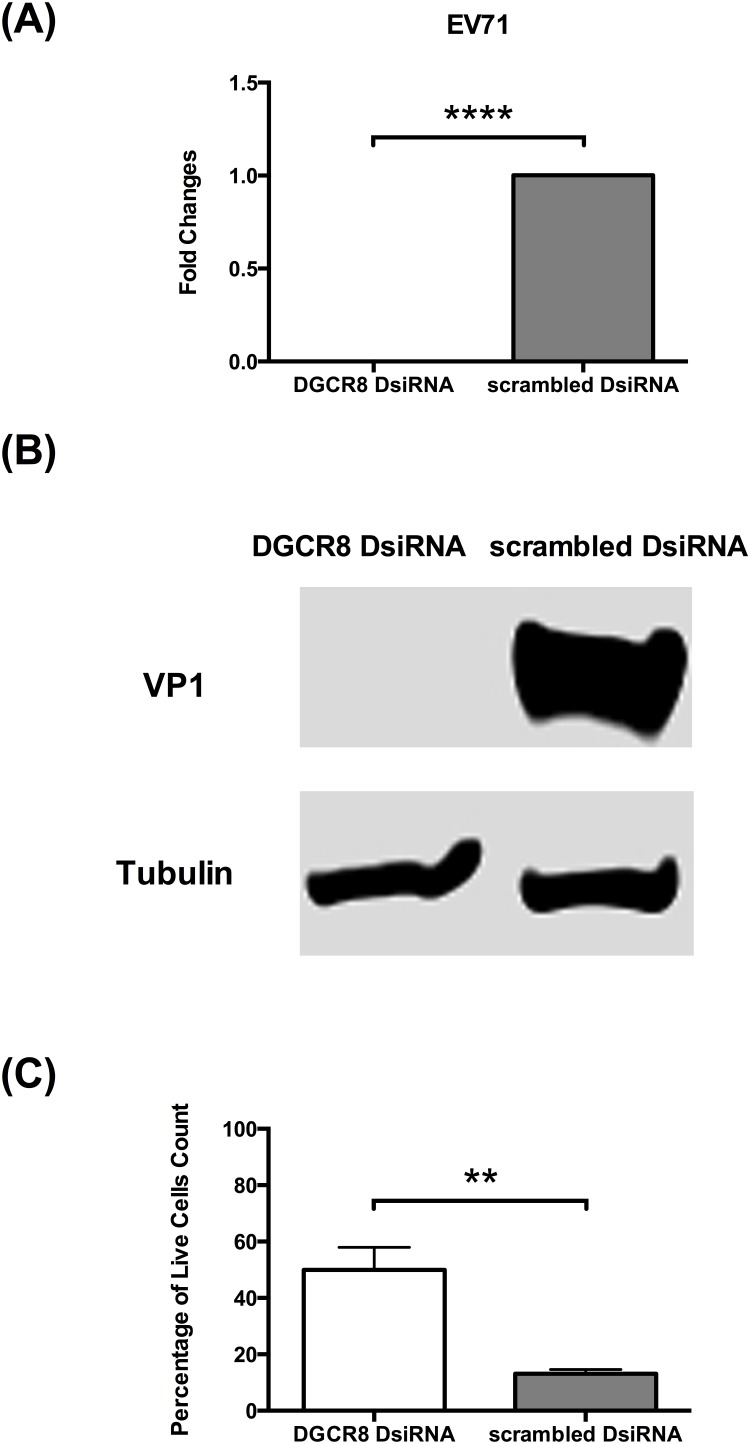
miRNA depleted HT29 cells is not able to support EV71 replication. DGCR8 knockdown HT29 cells and control HT29 cells (scrambled DsiRNA) were infected with EV71 (MOI of 1). Total intracellular RNA were harvested at 36****hpi, converted to cDNA and measured by qPCR with primers specific to viral VP1. Total intracellular protein were harvested at at 36****hpi and measured by western blot (A) Relative expression of EV71 viral RNA measured by qPCR. (n = 3, **** = *p* values of <0.0001) (B) Western blot analysis for EV71 VP1 viral protein. (C) Cell viability assessed at 72****hpi using vital dye trypan blue. (n = 3, ** = p values of <0.01).

**Figure 3:**
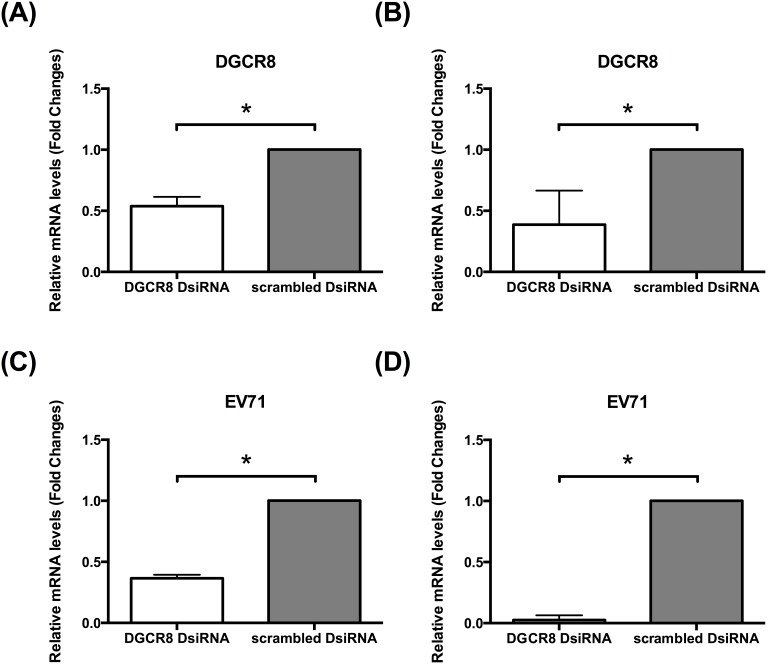
EV71 is unable to establish infection in both RKO and RD miRNAs depleted cells. DGCR8 knockdown cells and control cells (scrambled DsiRNA) in both RKO and RD cell line were infected with EV71 (MOI of 1). Total intracellular RNA were harvested at 12****hpi, converted to cDNA and measured by qPCR with primers specific to viral VP1. (A) Relative expression of DGCR8 gene in miRNAs depleted RKO cells measured by qPCR verifying knockdown. (n = 3, * = *p* values of <0.05) (B) Relative expression of DGCR8 gene in miRNAs depleted RD cells measured by qPCR verifying knockdown. (n = 3, * = *p* values of <0.05) (C) Relative expression of EV71 viral RNA in miRNAs depleted RKO cells measured by qPCR. (n = 3, * = *p* values of <0.05) (D) Relative expression of EV71 viral RNA in miRNAs depleted RKO cells measured by qPCR. (n = 3, * = *p* values of <0.05).

To investigate the role of miRNAs in EV71 infection, we compare EV71 replication in miRNAs depleted HT29 cells against control cells (scrambled DsiRNA). Using established protocols, the kinetics of EV71 RNA synthesis in both infected DGCR8 knockdown cells and control cells were examined quantitatively using real time quantitative polymerase chain reaction (qPCR) at 36****h post infection (hpi). Interestingly, DGCR8 knockdown (miRNAs depleted) HT29 cells were unable to support EV71 proliferation compared to control cells (Figure 2). There is a significant decrease in viral RNA between DGCR8 knockdown HT29 cell in contrast with control cells (scrambled DsiRNA) (Figure 2a). Consistent with our qPCR analysis, protein analysis of viral VP1 protein representation of EV71 replication, were shown to be highly downregulated in EV71 infected DGCR8 knockdown cells (Figure 2b). To further elucidate the effect of miRNAs during EV71 infection, we asked if the host cells survivability was enhanced in EV71 infected miRNAs depleted cells. Indeed, it was observed that at 72 hpi, there was a statistically significant percentage of living cells in EV71 infected DGCR8 knockdown cells compared to control cells infected with EV71 (Figure 2c and Figure S1). In order to validate our findings in HT29 cells, we further test this on another colorectal cell line (RKO) and skeleton muscle cell line (RD) where we knock down DGCR8 and examine the effect on EV71 replication (Figure 3). Consistent with our findings in HT29 model, EV71 is unable to establish infection in miRNAs depleted cells (HT29, RKO and RD cells) (Figure 2 and 3).

Taken together, DGCR8 or rather miRNAs affects EV71 replication and recused its detrimental phenotype.

### Identification of deregulated miRNAs during EV71 infection

**Figure 4:**
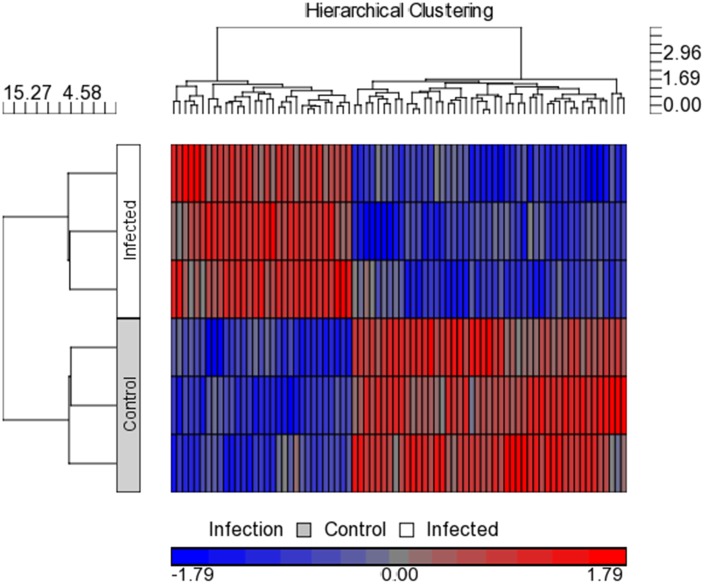
Heat map of the microarray miRNA expression profile of EV71 infected and non-infected control colorectal cells, HT29. The two-way hierarchical cluster heat map showed differential expressed miRNAs of two groups of samples. The miRNAs were chosen according to the cut off p<0.05 where blue represents miRNAs with decreased expression and red represent miRNAs with increased expression. (n = 3, * = p values of <0.05).

**Figure 5:**
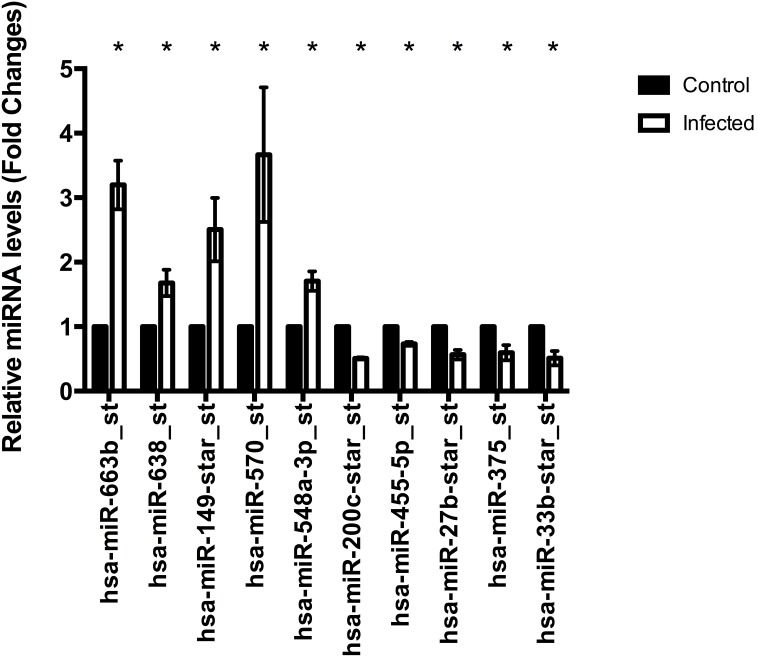
Validation of differential expression of selected miRNAs by qPCR. Ten expression levels of selected miRNAs from microarray assay were validated using qPCR. U6snRNA was used as reference RNA for the normalization of miRNAs and relative gene expression was quantified based on 2^−ΔΔCT^. (n = 3, * = p values of <0.05).

**Figure 6:**
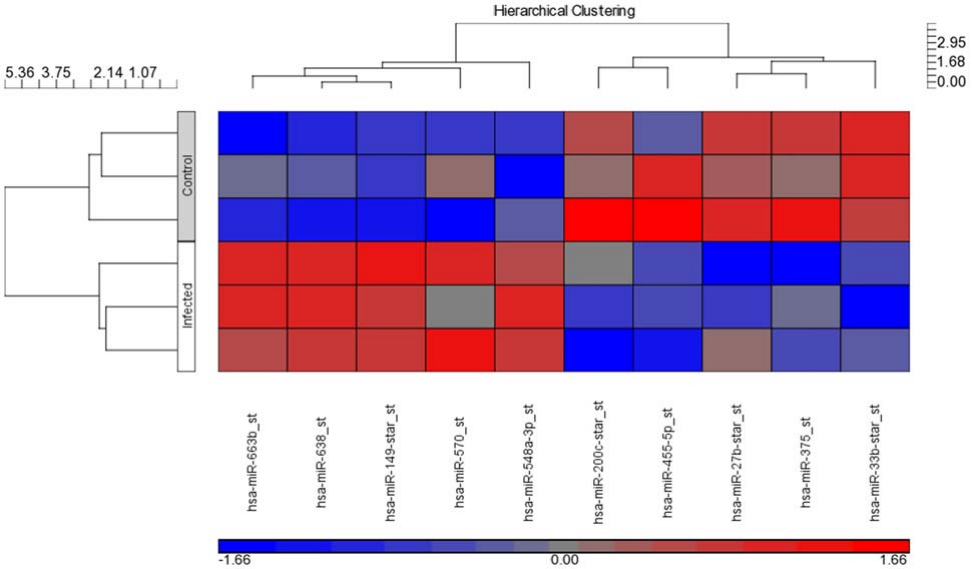
Heat map of differential expression of selected miRNAs by microarray. The two-way hierarchical cluster heat map showed differential expressed selected miRNAs of two groups of samples. The miRNAs from both methodology qPCR data showed similar direction of response in microarray analysis data with both methodologies showing similar trends where blue represents miRNAs with decreased expression and red represent miRNAs with increased expression.

To pinpoint on the differentially transcribed miRNAs used by EV71 during infection, we perform miRNAs profiling using Affymetrix GeneChip miRNA array. Specifically, comparative miRNAs expression between EV71 infected cells and control non-infected cells were performed. Microarray hybridisation identified 78 miRNAs being differentially expressed during EV71 infection (p<0.05) (Figure 4 and Table S1). To confirm the microarray results, relative abundance of the selected miRNAs were assayed using qPCR. The expression pattern from 10 selected miRNAs qPCR data showed similar direction of response in microarray analysis data with both methodologies showing similar trends (Figure 5 and 6). This indicates high quality and reliability of the microarray data analysis.

### EV71 down regulate host immune system using host miRNAs during infection

**Figure 7:**
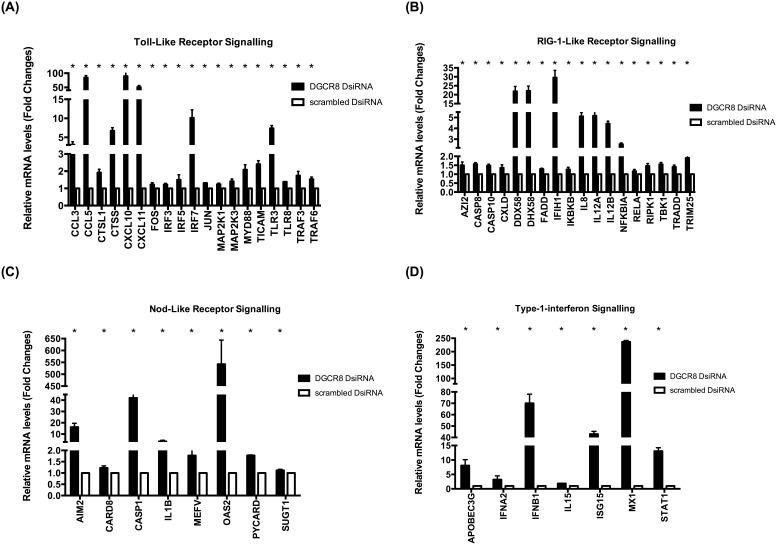
EV71 is unable to down regulate host antiviral response in miRNA depleted cells during infection. DGCR8 knockdown cells and control cells (scrambled DsiRNA) were infected with EV71 (MOI of 1). Total intracellular RNA were harvested at 36****hpi, converted to cDNA and measured by quantitative real time polymerase chain reaction (qPCR) (n = 3, * = p values of <0.05). A) Relative expression of Toll-like receptor signalling measured by qPCR. (B) Relative expression of RIG-1 like receptor signalling measured by qPCR. (C) Relative expression of Nod-like receptor signalling measured by qPCR. (D) Relative expression of Type 1 interferon signalling measured by qPCR.

To understand why miRNAs depleted cells resulted in the inability of EV71 to established infection; we examine the expression of 84 key genes involved in the innate antiviral immune response between EV71 infected miRNAs depleted HT29 cells in comparison with control cells (scrambled DsiRNA). In particular, we examine the host antiviral expression profile between infected DGCR8 knockdown cells against control cells. Using qPCR, quantitative expression of 52 antiviral genes from various antiviral signalling pathways such as Toll-like receptor signalling, Nod-like receptor signalling, RIG-1-like receptor signalling and type 1 interferon signalling were statistically up regulated in DGCR8 knockdown cells (Figure 7a, 7b, 7c, 7d). Noteworthy, CASP1, OAS2, DDX58, DHX58, CCL5, CXCL10, CXCL11, IRF7, IFBB1, ISG15, MX1 and STAT1 have at least 10–550 fold increase (Figure 7a, 7b, 7c, 7d).

## Discussion

Viruses have developed highly sophisticated gene-silencing mechanisms to evade host-immune response in order to establish infection. One of such mediators used by viruses is miRNAs. miRNAs is a new paradigm of RNA-directed gene expression regulation which contribute to the repertoire of host-pathogen interactions during viral infection. Cellular miRNAs could be involved during viral infection with either positive or negative effects on viral replication kinetics [36], [37]. Indeed, computational prediction reveal that one miRNAs may regulate hundreds of genes while one gene may be in turn regulated by hundreds of miRNAs [38]. However, the role of cellular miRNAs in the defence against viral infection has thus remained elusive. Given the scope of miRNA-mediated gene regulation in the mammalian system, cellular miRNAs may directly or indirectly affect virus pathogenesis. Cellular miRNA expression could be remodelled by viral infection, which can alter the host cellular microenvironment and attributing to both host antiviral defence and viral factors [25], [32], [36], [39], [40].

The biogenesis of miRNAs begins with the transcription of a long primary transcript known as pri-miRNA by RNA polymerase II [27]–[29], [32], [39], [41]. Pri-miRNAs are cleaved/processed by RNase III enzyme (Drosha), double-stranded-RNA-binding protein, (DiGeorge Syndrome Critical Region; DGCR8) and RNAse III-like Dicer to become functional miRNAs. In this study, we have demonstrated that abolishing functional miRNAs in human epithelial colorectal cell line (HT29) impair EV71 replication while this was not observed in control cells treated with scrambled DsiRNA. On the contrary, global knockdown of miRNAs have been previously shown to have enhance HIV-1 infection [42], [43]. Knock down of DGCR8, a precursor to miRNAs biogensis were shown to enhance human immunodeficiency virus (HIV) replication [42], [43]. This implies that EV71 utilise miRNAs during infection and in the absent of miRNAs, EV71 was unable to successfully mount the infection.

Another study reported by Otsuka and colleagues showed a similar finding in Vesicular stomatitis virus (VSV) infection [44]. Otsuka and colleagues observed that impaired miRNA production in mice with dicer deficient mice resulted in the hypersensitivity toward infection by VSV [44]. It was observed there is no alternation of interferon-mediated antiviral response by dicer deficiency instead the change in virus replication is due to miR-24 and miR-93 [44]. Otsuka and colleagues shown that miR-24 and miR-93 could target VSV viral protein and thus in miRNA depleted cells, VSV replication was enhanced in the absence of miR-24 and miR-93 [44].

In recent years, there are reported evidences of specific host-cellular miRNAs modulating host-pathogen interaction [44]–[51]. Cytomegalovirus (CMV) for example was observed to down regulate miR-27a [45]. Overexpression of miR-27a using miRNAs mimics was shown to inhibit CMV proliferation and virus titres suggesting that miR-27a or miR-27a-regulated genes plays a role during CMV infection [45], [52]. Hepatitis C virus (HCV), an enveloped RNA virus of the Flavivirus family, responsible for both acute and chronic hepatitis in humans induced a liver-specific cellular miRNA, miR-122 for the facilitation of HCV replication [17], [48], [51], [53]–[55]. It was suggested that miR-122 is a valuable target for antiviral intervention and therapeutics. *In vivo* treatment of HCV-infected chimpanzees with antisense miR-122 successfully reduced HCV virus titres demonstrating promising antiviral treatment against virus induced diseases [48], [49], [53], [54].

Indeed, various miRNAs have been identified to play a role during EV71 pathogenesis. Recent publications of investigations into the role of miRNA in enterovirus infection have reported that enterovirus-induced miRNAs such as miR-23b, miR-296-5p, miR-141 and miR-146a play a role in EV71 replication [8], [56]–[58]. The use of miRNA inhibitors or mimics have demonstrated that miRNAs plays an important role in EV71 pathogenesis. miRNA inhibitors or mimics on these miRNAs either inhibited or enhanced EV71 replication during infection.

In particular, our results revealed miR-548 to be significantly up regulated in EV71 infected cells at 36****hpi in comparison with control non infected cells. Further bioinformatics analysis revealed that miR-548 was involved in antiviral response during infection [17], [23], [25], [38], [59]. Consistent with our findings, Li and colleagues have recently identified the involvement of miR-548 in IFN signalling pathways during viral infection [59]. Li and colleagues demonstrated that miR-548 down-regulates host antiviral response via direct targeting of IFN-λ1 [59]. In contrast, in EV71 infected rhabdomyosarcoma (RD) cells, miR-548 was observed to decrease in a time dependent manner [59]. In view that the host innate system is the first line of defence with IFNs being a key player during viral infection, it is atypical that miR-548 was up regulated to down regulate the host immune system [17], [18], [20]–[25]. The use of miR-548 mimics and inhibitors were shown to enhance or inhibit EV71 replication in RD cells respectively [59].

The innate immune system has evolved an arsenal of mechanism to sense and destroy invading pathogen. An interesting question that arises is whether EV71 is able to regulate host cellular miRNA transcription and processing during infection thereby leading to the down-regulation of deleterious cellular miRNA and/or the up-regulation of advantageous cellular miRNAs to enhance its replication in the host cells. To add to this intriguing interplay between cellular miRNAs and viral pathogens, Huang and colleagues has demonstrated that HIV, an enveloped RNA virus of the Retreoviridae family utilise host cellular miR-28, miR-125b, miR-150, miR-223 and miR-382 to control viral protein synthesis in order to evade host immune system [47]. Epstein-Barr virus (EBV), a DNA virus of the Herpes family, for example induce the expression of miR-29b, miR-155 and miR-146a to mediate the down regulation of innate immune response and IFN signalling pathways to enhances its survival [46], [60], [61]. Lecellier and colleagues have shown that in primate foamy virus type 1 (PFV-1), overexpression of cellular miRNA miR-32 could induce an antiviral response to inhibit virus replication [37].

**Figure 8:**
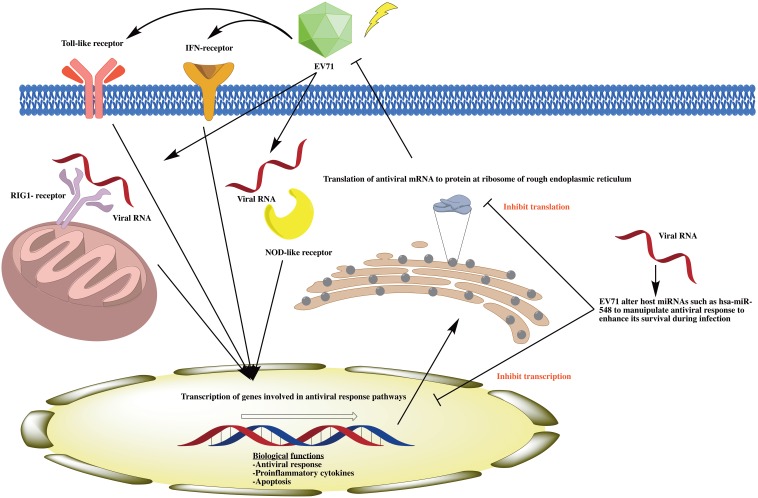
Schematic illustration of the interplay between EV71 and host miRNAs. EV71 altered host miRNAs such as has-miR-548 to manipulate host antiviral responses such as Toll-like signalling, NOD-like signalling, RIG-1 signalling and Type I interferon pathways to enhance its survival during pathogenesis.

To test this reasoning that EV71 down regulate host miRNAs during infection to enhance its survival, we next ask if the innate response and signalling pathways were altered in EV71 infected miRNA-depleted cells. Therefore, we analysed the expression of 84 key genes involved in the innate antiviral immune response between EV71 infected miRNAs depleted HT29 cells in comparison with control cells (scrambled DsiRNA). Quantitative analysis\revealed 52 antiviral genes from various antiviral signalling pathways such as Toll-like receptor signalling, Nod-like receptor signalling, RIG-1-like receptor signalling and type 1 interferon signalling were significantly up regulated in EV71 infected miRNAs depleted HT29 cells. In particular, CASP1, OAS2, DDX58, DHX58, CCL5, CXCL10, CXCL11, IRF7, IFBB1, ISG15, MX1 and STAT1 have at least 10–550 fold increase. The enhanced host antiviral response in miRNA-depleted cells during EV71 infection may result in the inability for EV71 to replicate during infection (Figure 8).

The host immune response is responsible for the eradication of invading pathogens, as such viruses has developed means to counteract with the induction, signalling and antiviral pathways [16], [20], [21], [25], [40]. RNA viruses generally block and inhibit IFN and antiviral molecules detrimental to virus replication [20], [24]. Despite all the sophisticated mechanism used by viruses to inhibit host immune system, host has been under evolutionary pressure against viral antagonism of IFN system [20], [21], [24], [40]. Our result suggests that EV71 attenuate host miRNAs to mediate host immune system during infection (Figure 8). As a result of DGCR8 knockdown, EV71 is unable to regulate host miRNAs to enhance cellular microenvironment leading to the inability to establish infection (Figure 8). Notwithstanding, IFN signalling pathways such as type I interferon was previously demonstrated to play an important role during EV71 pathogenesis [62], [63]. These results warrant further studies for the identification of specific miRNAs which play a pivotal role during EV71 pathogenesis specifically targeting the host antiviral signalling pathway.

## Conclusion

In conclusion, we provided evidence for an intricate physiological interplay between host miRNAs machinery and invading pathogen, EV71. We reported three salient findings. First, DGCR8, an essential cofactor for microRNAs biogenesis contribute to a central role during EV71 pathogenesis. We have shown that EV71 is not able to replicate efficiently in miRNAs depleted cells. Secondly, our results suggest that EV71 may remodel the cellular miRNAs composition thereby utilising the host cellular miRNAs to enhance cellular microenvironment for viral replication. Specifically, our result suggests that EV71 may be at least partially responsible for the up regulation of host miR-548 to enhance cellular microenvironment for viral replication. Thirdly, remodelling of miRNAs during EV71 infection plays a significant role specifically in antiviral response. We have demonstrated that in EV71 infected miRNAs depleted cells, host antiviral responses from various antiviral signalling pathways such as Toll-like receptor signalling, Nod-like receptor signalling, RIG-1-like receptor signalling and type 1 interferon signalling were significantly higher in comparison with EV71 infected control cells. This may explain our result on the inability for EV71 to infect miRNAs depleted cells given the enhanced host antiviral response. Taken together, our study has provide valuable knowledge toward the study of host-pathogen interaction during EV71 pathogenesis and this could lead to future investigation for the development of antiviral therapeutics using miRNAs inhibitors against EV71. This will open the door toward novel host-defence mechanism that exists in mammalian cells as well as to the antiviral mechanisms employed during EV71 pathogenesis.

## Materials and Methods

### Cell culture and virus propagation

The EV71 strain used in this study was isolated from a fatal case of HFMD during October 2000 outbreak in Singapore, Enterovirus 5865/sin/000009 strain from subgenogroup B4 (accession number 316321; hereby designated as Strain 41) (a gift from Mrs Phoon Meng Chee, Department of Microbiology, National University of Singapore). Human colorectal cell line (HT29) (ATCC catalog no. HTB-38) was maintained in Roswell Park Memorial Institute medium (RPMI) (PAA Laboratories, Austria) supplemented with 10% (v/v) Fetal Bovine Serum (FBS) (PAA Laboratories, Austria) and 2% penicillin–streptomycin (PAA Laboratories, Austria) at 37°C with 5% CO_2_. Human skeleton muscle cell line (RD) (ATCC catalog no. CCL-136) and Human colorectal cell line (RKO) (ATCC catalog no. CRL-2577) were maintained in Eagle’s minimum essential medium (EMEM) (PAA Laboratories, Austria) supplemented with 10% (v/v) Fetal Bovine Serum (FBS) (PAA Laboratories, Austria) and 2% penicillin–streptomycin (PAA Laboratories, Austria) at 37°C with 5% CO_2_. The virus stock was prepared by propagation of viruses using 90% confluent HT29 cells monolayer in RPMI with 10% FBS at 37°C with 5% CO_2_. The virus titres were determined using 50% tissue culture infective dose (TCID_50_) per millilitre (mL) according to Reed and Muench method [64].

### DsiRNA transfection and EV71 infection

HT29, RD and RKO cells were seeded at a concentration of 2×10^5^ cells/ml in 6-well plates and incubated for 8 h at 37°C with 5% CO_2_. Cells were washed twice with phosphate buffered saline (PBS) and transfected using Lipofectamine RNAiMAX (Invitrogen Corporation, CA, USA) with TriFECTa Dicer-Substrate RNAi KIT (Integrated DNA Technology, Coralville, IA, USA). Briefly, 10****nM of DGCR8 DsiRNA (NM_001190326 duplex 2), NC1, negative control duplex-scrambled DsiRNA (NM_001190326) and TYE 563 DS Transfection Control, fluorescent-labeled transfection control duplex (NM_001190326) were used to transfect the cells respectively (Integrated DNA Technology, Coralville, IA, USA). Following 24 h after transfection, cells were washed twice with PBS and infected with EV71 at multiplicity of infection (MOI) of 1. The culture media were removed and replaced with 2****mL of fresh RPMI medium after 1 h of incubation at 37°C with 5% CO_2_.

### EV71 infection for miRNA profiling

HT29 cells were seeded at a concentration of 5×10^5^ cells/ml in 6-well plates and incubated for 24 h at 37°C with 5% CO_2_. Cells were washed twice with phosphate buffered saline (PBS) and infected with EV71 at multiplicity of infection (MOI) of 1 or nil respectively. The culture media were removed and replaced with 2 mL of fresh RPMI medium after 1 h of incubation at 37°C with 5% CO_2_.

### Total cellular RNA extraction

The respective infected cells were harvest at 12 or 36 hours posts infection (hpi) respectively. The total cellular RNA of HT29, RD and RKO cells were extracted using the miRNeasy mini kit (Qiagen, Hilden, Germany) in accordance to the manufacturer’s instructions. Briefly, the cells were lysed and homogenise using lyses solution provided (Qiagen, Hilden, Germany). Total RNA were harvested using the RNeasy spin column and wash twice before elution (Qiagen, Hilden, Germany). Harvested total RNA was quantitated using Nanodrop 100 spectrophotometer (ThermoScientific, Waltham, USA).

### miRNA microarray analysis

The extracted total RNA was labelled with FlashTag Biotin HSR RNA Labeling Kits for the Affymetrix GeneChip miRNA array according to manufacturer’s instruction. Briefly, one microgram of total RNA will be incubated with ATP and Poly A polymerase at 37°C for 15****m to add a 3′ polyA tail. A ligation reaction will then be performed to covalently attach to the miRNA population a multiple-biotin molecule containing a 3DNA dendrimer. Labelled samples will subsequently be processed according to manufacturer’s instructions for the Affymetrix miRNA Array 2.0 (Affymetrix, Santa Clara, CA). After hybridisation for 16 h at 48°C, the arrays will be washed and stained in an Affymetrix Fluidics station 450, then scanned in an Affymetrix 3000 7G scanner. Raw data for the microarray (GSE57372) was analysed with Partek Genomics Suite (Partek Incorporated, Saint Louis, USA) where p<0.05 is considered significant.

### cDNA synthesis

For the conversion of mRNA to cDNA, 1 µg of the total RNA was reverse transcripted using RT^2^ First Strand kit (Qiagen, Hilden, Germany) in accordance to the manufacturer’s instructions. Briefly, 1 µg of the extracted RNA was first mixed with Buffer GE to a total volume of 10 µl for genomic DNA elimination and incubated at 42°C for 5 m and on ice for 1 m. Reverse-transcription mix which consist of enzyme reverse transcriptase and buffer was then added to a volume of 20 µl and subjected to thermal profile of 42°C for 15 m followed by 95°C for 5 m. 91 µl of RNase free water was then added to each reaction in accordance to the manufacturer’s instructions.

For conversion of microRNA to cDNA, 10 ng of the total RNA was reverse transcripted using the Universal cDNA Synthesis kit (Exiqon, Denmark) the manufacturer’s instructions. Briefly, 10****ng of the extracted RNA will be mixed with enzyme reverse transcriptase and buffer to a volume of 10 µl. The reaction mix will then subject to thermal profile of 25°C for 5 m, 42°C for 30 m followed by 85°C for 5 m according to the manufacturer’s instructions.

### Quantitative real time polymerase chain reaction

The EV71 specific primers targeting the conserve VP1 regions were 5′-GCTCTATAGGAGATAGTGTGAGTAGGG-3′ and the reverse primer 5′-ATGACTGCTCACCTGCGTGTT-3′ [65]. Primers for DGCR8 were 5′-ACTTGTGCATGTTAGCTGTGTAGA-3′ and the reverse primer 5′-GCTTAAGACTAGTTTACAAGACCAGAGT-3′ were designed to span exon-exon boundaries. The primers for the house keeping gene actin (β-ACT) used were 5′-ACCAACTGGGACGACATGGAGAAA-3′ and the reverse primer 5′-TAGCACAGCCTGGATAGCAACGTA-3′. The qPCR was performed using the iTaq Universal SYBR Green Supermix (Bio-Rad Laboratories, CA, USA) on the BioRad CFX96 Real-Time PCR system (Bio-Rad Laboratories, CA, USA). Briefly, 1 µl of cDNA and 1 µl of the forward and the reverse primers were added iTaq Universal SYBR Green Supermix. The reaction mix was then subjected to thermal profile of denaturation at 95°C for 30****s, followed by amplification and quantification in 40 cycles at 95°C for 5****s followed by 60°C for 30 s. At the end of amplification cycles, melting temperature analysis was performed by the BioRad CFX96 Real-Time PCR system (Bio-Rad Laboratories, CA, USA). Relative gene expression was quantified based on 2^−ΔΔCT^ method [66].

For RT^2^ profiler PCR array, PCR arrays were performed with customized PCR containing pre-dispensed primers to monitor Human antiviral response PAHS-122ZD-2 (Qiagen, Hilden, Germany). qPCR was performed using RT^2^ SYBR Green qPCR mastermix (Qiagen, Hilden, Germany). Briefly, a total volume of 25 µl of cDNA and RT^2^ SYBR Green qPCR mastermix were added into each well of the Human antiviral response array 96 wells plate PAHS-122ZD-2 (Qiagen, Hilden, Germany) in accordance to the manufacturer’s instructions. The reaction mix was then subjected to thermal profile of denaturation at 95°C for 10****m, followed by amplification and quantification in 40 cycles at 95°C for 15 s followed by 60°C for 1****m. At the end of amplification cycles, melting temperature analysis was performed by the BioRad CFX96 Real-Time PCR system (Bio-Rad Laboratories, CA, USA).

For the detection of microRNA, qPCR was performed using the ExiLENT SYBR Green Master mix (Exiqon, Denmark) on the BioRad CFX96 Real-Time PCR system (Bio-Rad Laboratories, CA, USA). Briefly, 2.5 µl of cDNA and 1 µl of the forward and the reverse primers was added to ExiLENT SYBR Green Master mix (Exiqon, Denmark) according to the manufacturer’s instructions. The reaction mix will then subjected to thermal profile of denaturation at 95°C for 10 m, followed by amplification and quantification in 40 cycles at 95°C for 10 s, 60°C for 30 s followed by 50°C for 30 s. At the end of amplification cycles, melting temperature analysis will be performed by the BioRad CFX96 Real-Time PCR system (Bio-Rad Laboratories, CA, USA). Primers for microRNA qPCR were designed and purchased from Exiqon, Denmark. U6snRNA was used as reference RNA for the normalization of microRNA (miRNA) qPCR data. Relative gene expression was quantified based on 2^−ΔΔCT^ method [66].

### Western blot

Total cellular protein for HT29 cells and control cells were collected using a lysis mix in mammalian cell lysis solution–CelLytic M (Sigma-Aldrich Pte Ltd, USA) in accordance with manufacturer’s instructions. Equal protein concentration (20 µg) from each samples were added onto 10% Mini-PROTEAN TGX (Bio-Rad Laboratories, CA, USA) and separated by electrophoresis. Separated proteins were transferred onto nitrocellulose membrane (Bio-Rad Laboratories, CA, USA). The membranes were incubated with mouse anti EV71 antibody (AbD serotech, Oxford, UK) or anti tubulin antibody (Santa Cruz Biotechnology inc, California, USA) respectively in shaker 4°C overnight. The membranes were washed three times in Tris-buffered saline with 0.05% tween-20 before and incubating with secondary antibodies conjugated to horseradish peroxidase (HRP) for 30****m. (ThermoScientific, Waltham, USA). After three wash with Tris-buffered saline and 0.05% tween-20, membrane was subjected to Clarity Western ECL Substrate (Bio-Rad Laboratories, CA, USA) in accordance with manufacturer’s instructions. The membranes were then scanned using the LI-COR C-DiGit Blot Scanner (LI-COR Biosciences, Lincoln, Nebraska, USA).

### Cell viability and counts

Cell count and viability was performed at 0 h, 48 h and 72 h on the Luna Automated Cell Counter system (Logos Biosystem, USA) in accordance to the manufacturer’s instructions. Briefly, the cells were trypsinised and topped up with fresh media to a total volume of 1000 µl of media and 10 µl of this cell suspension were mixed with 10 µl of tryphan blue. 10 µl of this diluted cell suspension were then loaded onto the Luna counting slide for analysis.

### Statistical analysis

All statistical analysis was performed on GraphPad Prism Version 6.0c (GraphPad Software, USA). Student *t* test was used to compare two groups. *p* values of <0.05 were considered statistically significant.

## Supporting Information

Figure S1 **Cell viability assessed 24 h after transfection and throughout 72 h post EV71 infection using vital dye trypan blue. **(n = 3, * = **p values of <0.05).**
(TIFF)

Table S1 **Altered miRNA expression profile of EV71 infected and non-infected control colorectal cells, HT29.**
(DOCX)
