# Supplementary figures and images for: Enterovirus71 (EV71) Utilise Host microRNAs to Mediate Host Immune System Enhancing Survival during Infection
Source: PLoS One. 2014 Jul 21;9(7):e102997. doi: 10.1371/journal.pone.0102997 (PMC4105423; doi:10.1371/journal.pone.0102997)

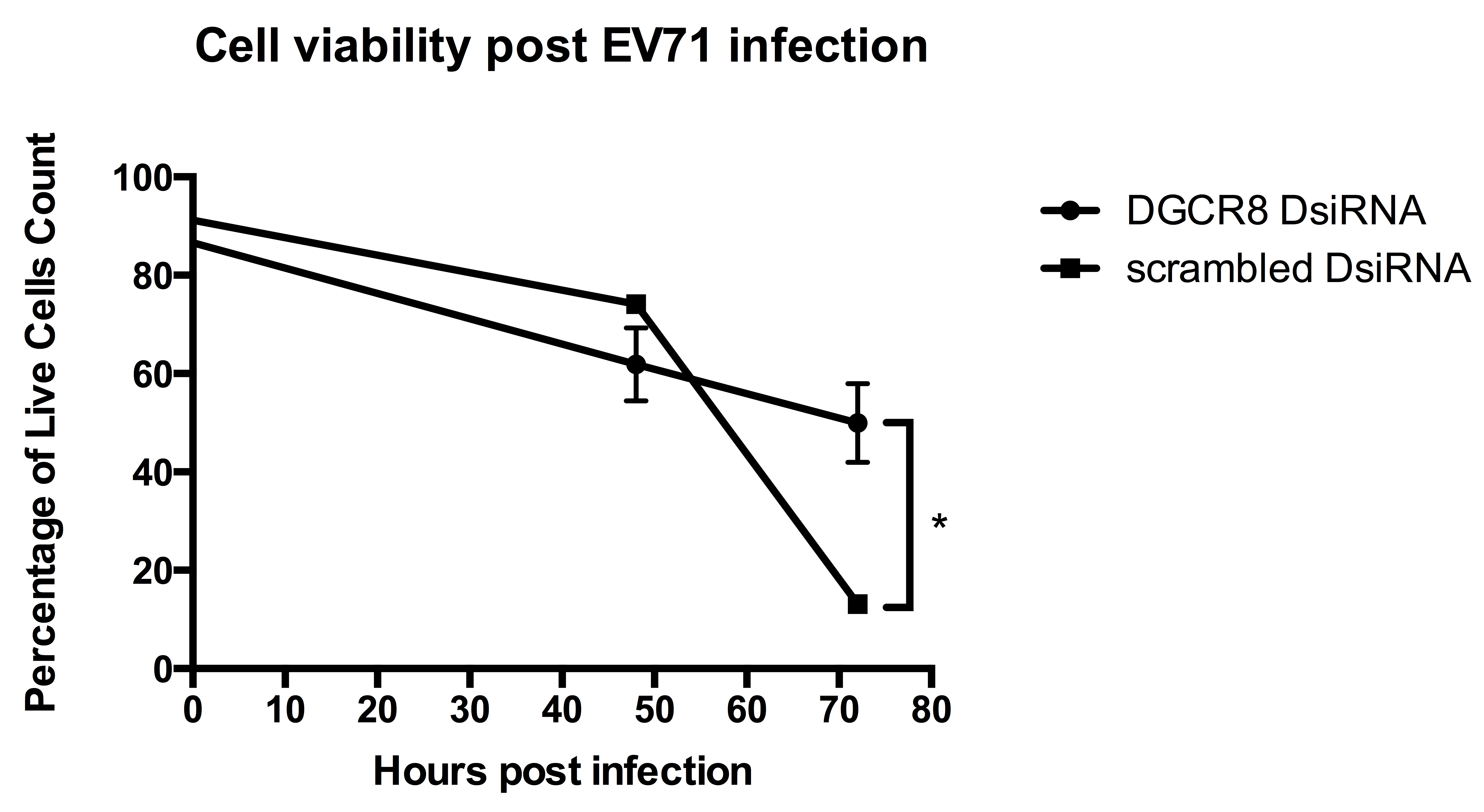

Supplement: Figure S1 — Cell viability assessed 24 h after transfection and throughout 72 h post EV71 infection using vital dye trypan blue. (n = 3, * = p values of <0.05). (TIFF) [file pone.0102997.s001.tif]
