# Supplementary material for: Enterovirus71 (EV71) Utilise Host microRNAs to Mediate Host Immune System Enhancing Survival during Infection
Source: PLoS One. 2014 Jul 21;9(7):e102997. doi: 10.1371/journal.pone.0102997 (PMC4105423; doi:10.1371/journal.pone.0102997)
Supplement: Table S1 — Altered miRNA expression profile of EV71 infected and non-infected control colorectal cells, HT29. (DOCX) [file pone.0102997.s002.docx]

**Supplementary Table 1. Altered miRNA expression profile of EV71 infected and non-infected control colorectal cells, HT29.**

| Column # | Probeset ID | Species Scientific Name | | p-value(Infected vs. Control) | Fold-Change(Infected vs. Control) |
| --- | --- | --- | --- | --- | --- |
| 11517 | hsa-miR-1975_st | Homo sapiens | 0.000163 | | 3.94209 |
| 11959 | hsa-miR-483-5p_st | Homo sapiens | 0.024704 | | 2.81949 |
| 12080 | hsa-miR-548a-3p_st | Homo sapiens | 0.010631 | | 2.71964 |
| 11669 | hsa-miR-3124_st | Homo sapiens | 0.047508 | | 2.50557 |
| 11434 | hsa-miR-149-star_st | Homo sapiens | 0.000677 | | 2.23921 |
| 12237 | hsa-miR-664-star_st | Homo sapiens | 0.01227 | | 2.22846 |
| 11617 | hsa-miR-2861_st | Homo sapiens | 0.002286 | | 2.059 |
| 12236 | hsa-miR-663b_st | Homo sapiens | 0.01177 | | 1.99451 |
| 19572 | v11_hsa-miR-768-3p_st | Homo sapiens | 0.000723 | | 1.89955 |
| 12166 | hsa-miR-601_st | Homo sapiens | 0.046423 | | 1.79713 |
| 12209 | hsa-miR-638_st | Homo sapiens | 0.002766 | | 1.78007 |
| 11499 | hsa-miR-1915_st | Homo sapiens | 0.001266 | | 1.70212 |
| 11710 | hsa-miR-3162_st | Homo sapiens | 0.027274 | | 1.56497 |
| 11874 | hsa-miR-4270_st | Homo sapiens | 0.002332 | | 1.47817 |
| 11486 | hsa-miR-1909-star_st | Homo sapiens | 0.024058 | | 1.46414 |
| 9845 | hp_hsa-mir-525_st | Homo sapiens | 0.017357 | | 1.45682 |
| 11288 | hsa-miR-1228-star_st | Homo sapiens | 0.044908 | | 1.3919 |
| 9062 | hp_hsa-mir-1181_st | Homo sapiens | 0.013386 | | 1.38869 |
| 11522 | hsa-miR-199a-3p_st | Homo sapiens | 0.008481 | | 1.34305 |
| 9576 | hp_hsa-mir-320c-2_x_st | Homo sapiens | 0.031207 | | 1.32182 |
| 9736 | hp_hsa-mir-449a_st | Homo sapiens | 0.025192 | | 1.29993 |
| 9165 | hp_hsa-mir-1282_st | Homo sapiens | 0.045774 | | 1.26961 |
| 9066 | hp_hsa-mir-1184-2_s_st | Homo sapiens | 0.019358 | | 1.2661 |
| 9507 | hp_hsa-mir-3154_st | Homo sapiens | 0.047258 | | 1.25549 |
| 10006 | hp_hsa-mir-620_x_st | Homo sapiens | 0.014759 | | 1.24735 |
| 9270 | hp_hsa-mir-15b_st | Homo sapiens | 0.001623 | | 1.23691 |
| 9136 | hp_hsa-mir-1263_st | Homo sapiens | 0.015698 | | 1.23327 |
| 11785 | hsa-miR-33a_st | Homo sapiens | 0.015369 | | 1.22434 |
| 9618 | hp_hsa-mir-372_st | Homo sapiens | 0.024112 | | 1.20353 |
| 10020 | hp_hsa-mir-631_x_st | Homo sapiens | 0.000101 | | 1.20226 |
| 9807 | hp_hsa-mir-516b-2_x_st | Homo sapiens | 0.013246 | | 1.20032 |
| 12294 | hsa-miR-922_st | Homo sapiens | 0.029909 | | -1.20845 |
| 11495 | hsa-miR-1913_st | Homo sapiens | 0.049946 | | -1.21026 |
| 11546 | hsa-miR-205_st | Homo sapiens | 0.007349 | | -1.21409 |
| 9598 | hp_hsa-mir-339_st | Homo sapiens | 0.019065 | | -1.21441 |
| 12204 | hsa-miR-633_st | Homo sapiens | 0.005741 | | -1.21803 |
| 11246 | hsa-miR-103-2-star_st | Homo sapiens | 0.000123 | | -1.22043 |
| 9174 | hp_hsa-mir-1288_st | Homo sapiens | 0.031884 | | -1.22398 |
| 11540 | hsa-miR-203_st | Homo sapiens | 0.011568 | | -1.22787 |
| 12172 | hsa-miR-607_st | Homo sapiens | 0.01372 | | -1.22914 |
| 9178 | hp_hsa-mir-1289-2_x_st | Homo sapiens | 0.041897 | | -1.22958 |
| 9707 | hp_hsa-mir-4304_st | Homo sapiens | 0.02584 | | -1.24091 |
| 11489 | hsa-miR-190b_st | Homo sapiens | 0.037805 | | -1.24125 |
| 12211 | hsa-miR-640_st | Homo sapiens | 0.014434 | | -1.24236 |
| 10128 | hp_hsa-mir-941-3_s_st | Homo sapiens | 0.001341 | | -1.2437 |
| 11763 | hsa-miR-323b-3p_st | Homo sapiens | 0.025807 | | -1.25967 |
| 12182 | hsa-miR-616-star_st | Homo sapiens | 0.03395 | | -1.26134 |
| 9895 | hp_hsa-mir-548i-4_st | Homo sapiens | 0.003498 | | -1.26684 |
| 11425 | hsa-miR-146b-5p_st | Homo sapiens | 0.008528 | | -1.26984 |
| 11822 | hsa-miR-375_st | Homo sapiens | 0.038667 | | -1.27214 |
| 12084 | hsa-miR-548c-3p_st | Homo sapiens | 0.014034 | | -1.27284 |
| 11797 | hsa-miR-34b_st | Homo sapiens | 0.01083 | | -1.27311 |
| 11672 | hsa-miR-3126-5p_st | Homo sapiens | 0.046993 | | -1.27517 |
| 12208 | hsa-miR-637_st | Homo sapiens | 0.039124 | | -1.28712 |
| 9214 | hp_hsa-mir-1321_st | Homo sapiens | 0.01516 | | -1.29294 |
| 11691 | hsa-miR-3144-3p_st | Homo sapiens | 0.04497 | | -1.30681 |
| 11578 | hsa-miR-219-5p_st | Homo sapiens | 0.003858 | | -1.31686 |
| 12087 | hsa-miR-548d-5p_st | Homo sapiens | 0.022997 | | -1.31845 |
| 9221 | hp_hsa-mir-133a-1_x_st | Homo sapiens | 0.044891 | | -1.32283 |
| 9307 | hp_hsa-mir-1911_st | Homo sapiens | 0.020344 | | -1.32376 |
| 12102 | hsa-miR-548t_st | Homo sapiens | 0.043826 | | -1.33336 |
| 11726 | hsa-miR-3178_st | Homo sapiens | 0.016713 | | -1.35363 |
| 11963 | hsa-miR-486-3p_st | Homo sapiens | 0.009167 | | -1.35607 |
| 12184 | hsa-miR-617_st | Homo sapiens | 9.54E-05 | | -1.35717 |
| 11458 | hsa-miR-181a-2-star_st | Homo sapiens | 0.047522 | | -1.35906 |
| 11770 | hsa-miR-329_st | Homo sapiens | 0.006891 | | -1.39187 |
| 11279 | hsa-miR-1208_st | Homo sapiens | 0.046593 | | -1.40286 |
| 12155 | hsa-miR-590-5p_st | Homo sapiens | 0.007432 | | -1.48358 |
| 11566 | hsa-miR-212_st | Homo sapiens | 0.036881 | | -1.52046 |
| 11473 | hsa-miR-185-star_st | Homo sapiens | 0.031342 | | -1.5402 |
| 11890 | hsa-miR-4286_st | Homo sapiens | 0.011371 | | -1.57105 |
| 11395 | hsa-miR-134_st | Homo sapiens | 0.011043 | | -1.66762 |
| 11331 | hsa-miR-1266_st | Homo sapiens | 0.035153 | | -1.6898 |
| 11256 | hsa-miR-10a-star_st | Homo sapiens | 0.015768 | | -1.74491 |
| 11960 | hsa-miR-484_st | Homo sapiens | 0.039047 | | -1.76997 |
| 11923 | hsa-miR-4317_st | Homo sapiens | 0.034233 | | -1.80619 |
| 11786 | hsa-miR-33b-star_st | Homo sapiens | 0.011657 | | -2.05341 |
| 11724 | hsa-miR-3176_st | Homo sapiens | 0.037357 | | -2.16789 |
